# Complete Response Following Multimodal Treatment of HER2-Positive Oligometastatic Apocrine Breast Carcinoma: A Report of a Rare Case and Literature Review

**DOI:** 10.7759/cureus.95374

**Published:** 2025-10-25

**Authors:** Souad Margoum, Soumiya Samba, Nourelhouda Mouhib, Ahmed BenSghier, Mohamed Moukhlissi, Soufiane Berhili, Loubna Mezouar

**Affiliations:** 1 Radiation Oncology, Faculty of Medicine and Pharmacy, Mohammed First University, Oujda, MAR

**Keywords:** apocrine breast carcinoma, breast cancer, her2-positive, multimodal treatment, oligometastatic disease, stereotactic ablative radiotherapy (sabr)

## Abstract

Apocrine carcinoma of the breast is a rare histologic subtype characterized by distinctive morphological and immunohistochemical features. Due to its infrequent occurrence, there is a paucity of specific management guidelines, with treatment generally following the protocols applied to more common breast cancer subtypes. Here, we report the case of a 54-year-old woman with HER2-positive apocrine breast carcinoma and a solitary bone metastasis in the L5 vertebra. She received neoadjuvant chemotherapy combined with dual anti-HER2 therapy (trastuzumab and pertuzumab), followed by a right mastectomy with axillary lymph node dissection, locoregional conformal radiotherapy, and stereotactic radiotherapy to the L5 lesion. The patient then underwent adjuvant maintenance targeted therapy. A complete response, both histological and metabolic, was achieved and has been sustained after two years of follow-up. This case underscores the value of a personalized, multimodal treatment approach.

## Introduction

Breast cancer remains the most frequently diagnosed cancer among women worldwide. It is also a leading cause of cancer-related mortality, ranking fifth in cancer deaths after lung, colorectal, liver, and stomach cancers [1]. Histologically, invasive ductal carcinoma of no special type (NST) accounts for about 70% of cases, while the remaining cases comprise less common variants [2]. One of the rarest subtypes is invasive apocrine carcinoma (IAC), which accounts for only 0.4% to 4% of all breast cancers [3].

This subtype is characterized by distinct apocrine morphological features on histopathology and typically lacks estrogen and progesterone receptors. Approximately one-third of tumors overexpress or amplify HER2 [4]. Invasive apocrine carcinoma mainly affects women over the age of 40, sharing a similar demographic profile with NST cases [5]. The risk factors are similar to those of other breast cancers and include nulliparity, late first pregnancy, early menarche, and late menopause. Some authors have hypothesized that prolonged exposure to sex hormones, particularly androgens, may contribute to apocrine metaplasia within breast tissue [6].

The presentation of apocrine breast carcinoma is characterized by its clinical polymorphism, exhibiting a broad spectrum of manifestations ranging from an absence of symptoms to the detection of a palpable, well-demarcated mass, occasionally accompanied by hemorrhagic nipple discharge or a cystic lesion, which can clinically resemble invasive ductal carcinoma [5,7]. The incidence of axillary lymph node involvement ranges from less than 1% to approximately 4%. However, the extant data in the literature remain extremely limited [8].

In this report, we present the case of a 54-year-old woman diagnosed with HER2-positive apocrine breast carcinoma in an oligometastatic setting. The therapeutic strategy consisted of neoadjuvant chemotherapy combined with dual HER2-targeted therapy, followed by mastectomy with axillary lymph node dissection, adjuvant radiotherapy, and maintenance therapy with trastuzumab and pertuzumab. Given the rarity of HER2-positive apocrine carcinoma and the scarcity of data on its optimal management, this report aims to contribute to the existing literature by highlighting the efficacy of a personalized, multimodal therapeutic approach in an oligometastatic context.

## Case presentation

**Figure 1:**
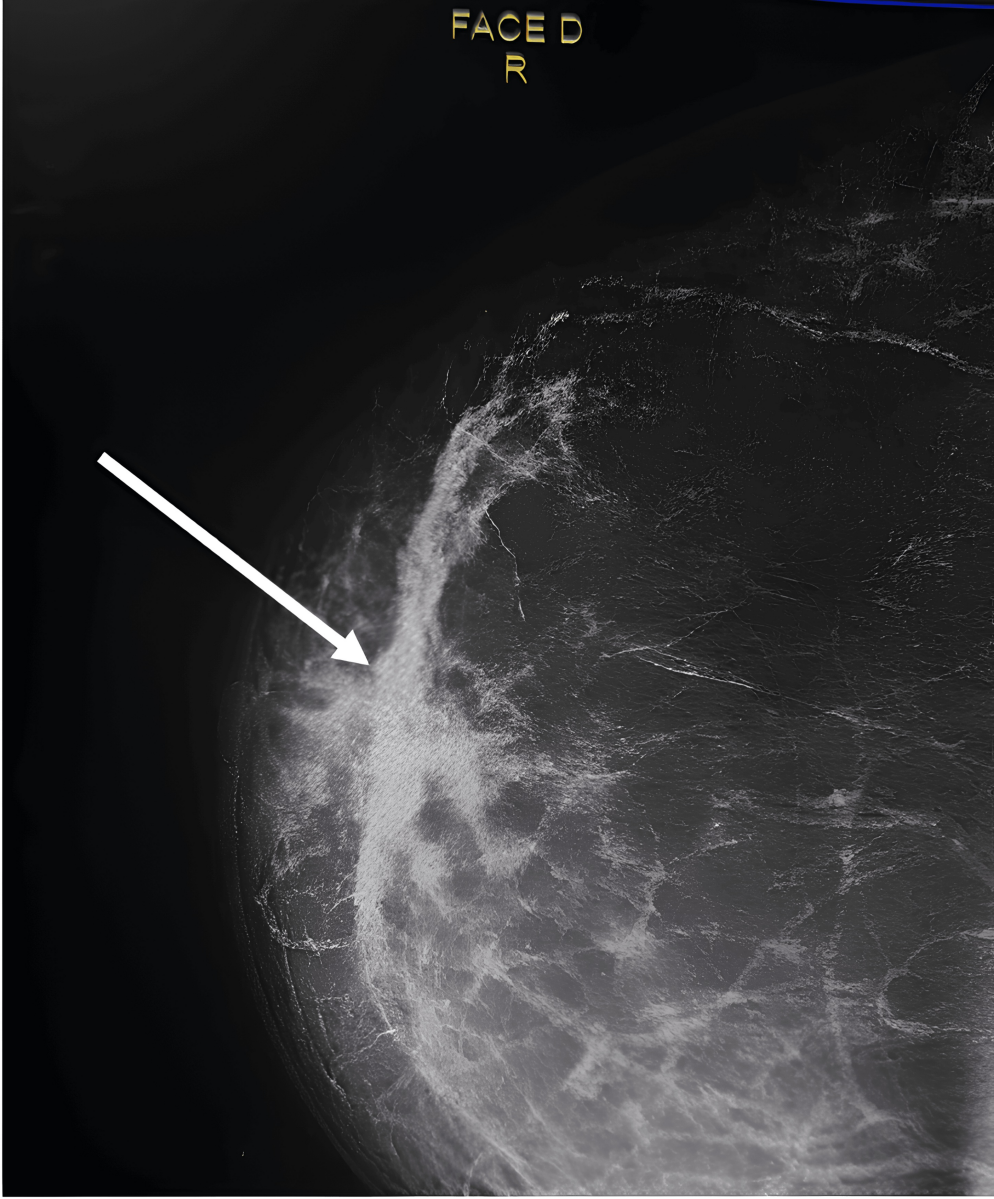
Mammogram (craniocaudal view) shows increased opacity in the form of upper and lower right retro-nipple plaques, with blurred contours and obscured by breast opacity (white arrow)

We report the case of a 54-year-old woman (G7P5) with an eight-year history of well-controlled hypertension and no personal or family history of breast cancer. Approximately nine months before presentation, she discovered a painless retroareolar nodule in her right breast during self-examination, with no associated mastodynia (breast pain) or nipple discharge. On clinical examination, a firm 3.5 cm mass was palpated behind the right areola, accompanied by localized erythema and fixed, non-tender ipsilateral axillary lymphadenopathy. Breast ultrasound and mammography revealed a suspicious retroareolar lesion with heterogeneous ductal ectasia, along with axillary lymph nodes classified as Breast Imaging-Reporting and Data System (BI-RADS) category 4 (Figure 1).

**Figure 2:**
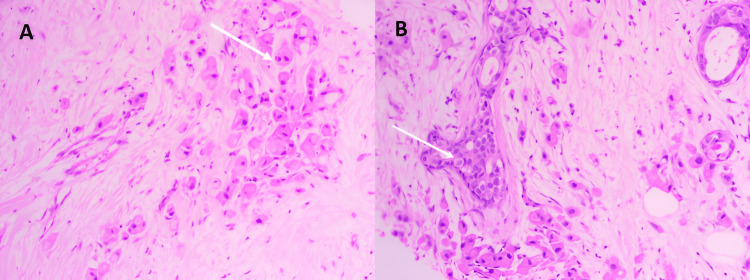
Histological images reveal a proliferation of atypical apocrine-type cells, characterized by abundant, granular, eosinophilic cytoplasm and hyperchromatic nuclei (white arrows)

Core needle biopsy confirmed IAC, Scarff-Bloom-Richardson grade II, with no in situ component and no lymphovascular invasion (Figure 2).

**Figure 3:**
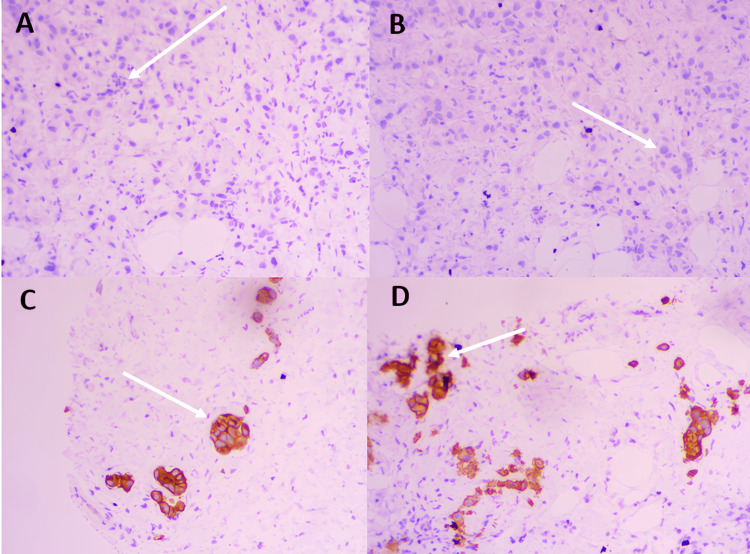
Immunohistochemical study A: PR with negative nuclear staining in tumor cells with positive internal control (white arrow); B: ER with negative nuclear staining in tumor cells with positive internal control (white arrow); C-D: HER2 intense and complete membrane staining in more than 90% of tumor cells (white arrows) PR: Progesterone receptor, ER: Estrogen receptor

Immunohistochemistry showed estrogen receptor (ER) negativity, progesterone receptor (PR) negativity, HER2 overexpression (3+), and a Ki-67 proliferation index of 25% (Figure 3).

**Figure 4:**
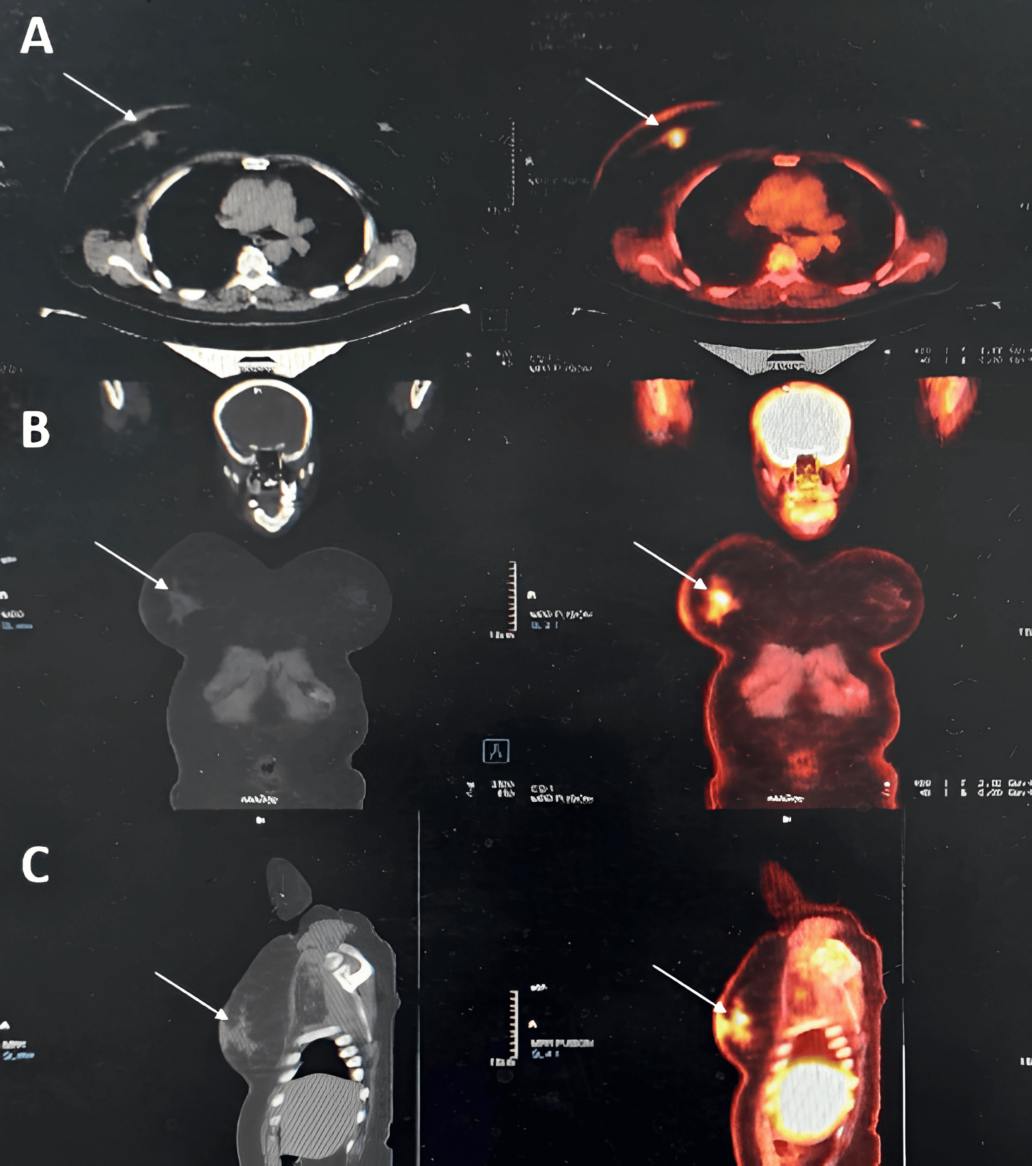
PET-CT images of the right breast Axial (A), coronal (B), and sagittal (C) sections show a hypermetabolic focus (white arrow) in the right breast located behind the nipple and corresponding to the known primary tumor.

**Figure 5:**
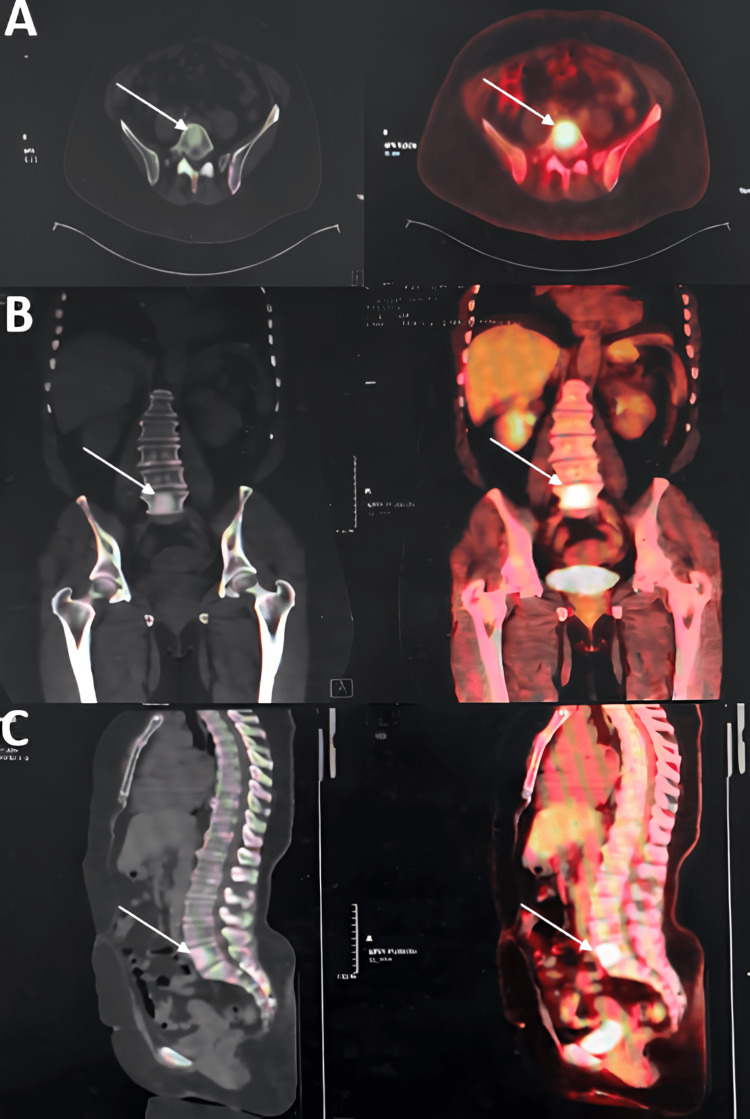
PET-CT images of the L5 vertebra Axial (A), coronal (B), and sagittal (C) sections show an osteocondensing and hypermetabolic lesion (white arrow) of the L5 vertebral body of secondary origin.

Staging with 18F-fluorodeoxyglucose (FDG) PET-CT demonstrated hypermetabolic foci in the retroareolar region (primary tumor) ​​​​​(Figure 4), the ipsilateral axillary lymph nodes, and a solitary sclerotic lesion in the L5 vertebra (Figure 5), without any visceral involvement. The disease was staged as T4b N2 M1 according to the tumor, node, and metastasis (TNM) classification.

Neoadjuvant therapy consisted of four cycles of dose-dense Adriamycin and Cytoxan (AC) (doxorubicin 60 mg/m² and cyclophosphamide 600 mg/m² every two weeks), followed by 12 weekly cycles of paclitaxel (80 mg/m²) in combination with trastuzumab (loading dose 8 mg/kg, then 6 mg/kg every three weeks) and pertuzumab (loading dose 840 mg, then 420 mg every three weeks). Post-treatment PET-CT showed complete metabolic resolution of the primary tumor, the axillary nodes, and the L5 lesion, with no new abnormalities.

**Figure 6:**
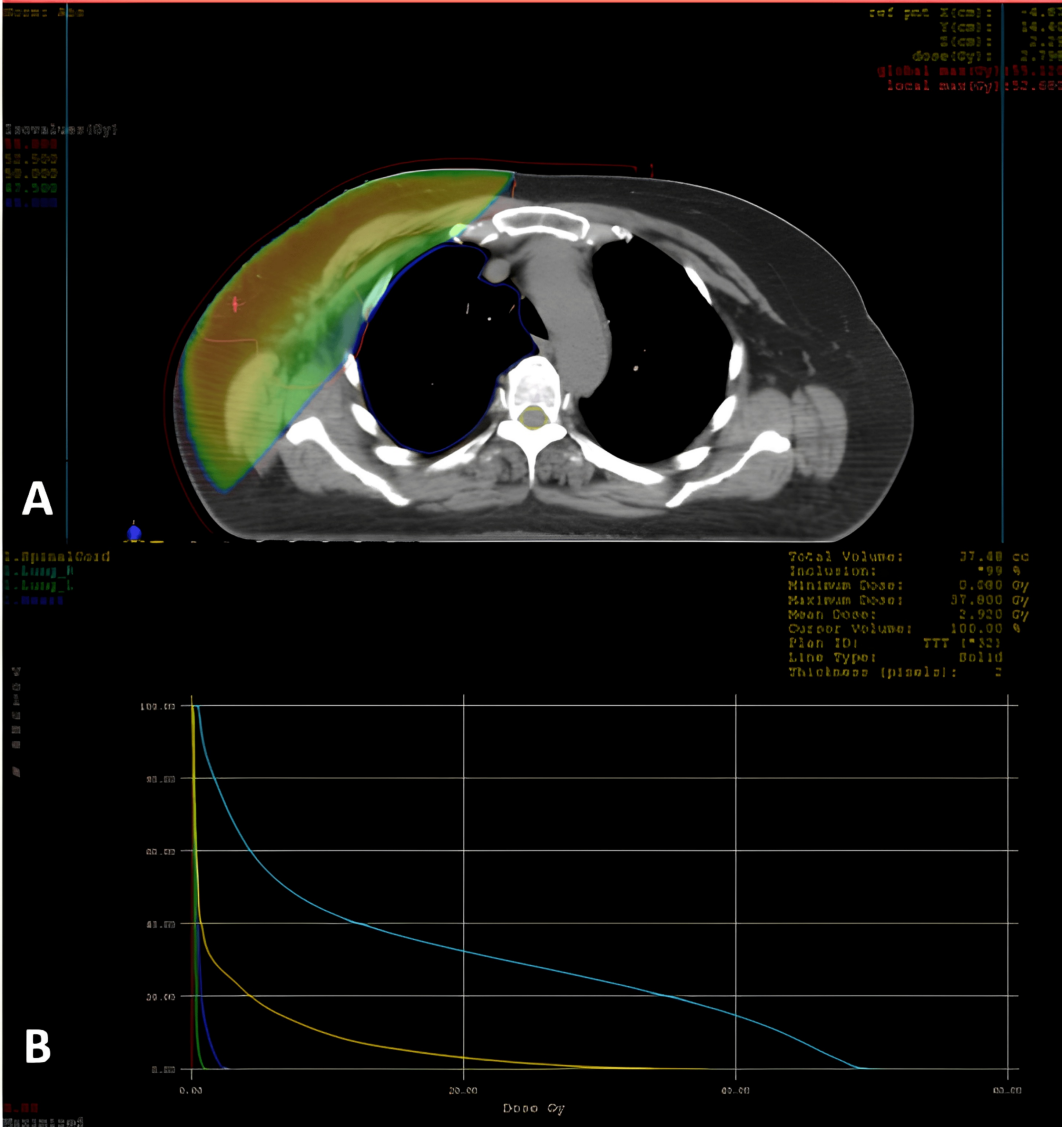
3D conformal radiotherapy of the right breast A: Axial slice of a dosimetric scan shows the distribution of isodoses; B: Dose–volume histogram (DVH) illustrates adequate target coverage and dose distribution to the organs at risk (lungs, heart, and spinal cord). The color-coded lines correspond to the critical structures. The dose scale is expressed in gray (Gy), with a maximum dose below 107% of the prescribed dose, ensuring optimal conformity and organ sparing.

**Figure 7:**
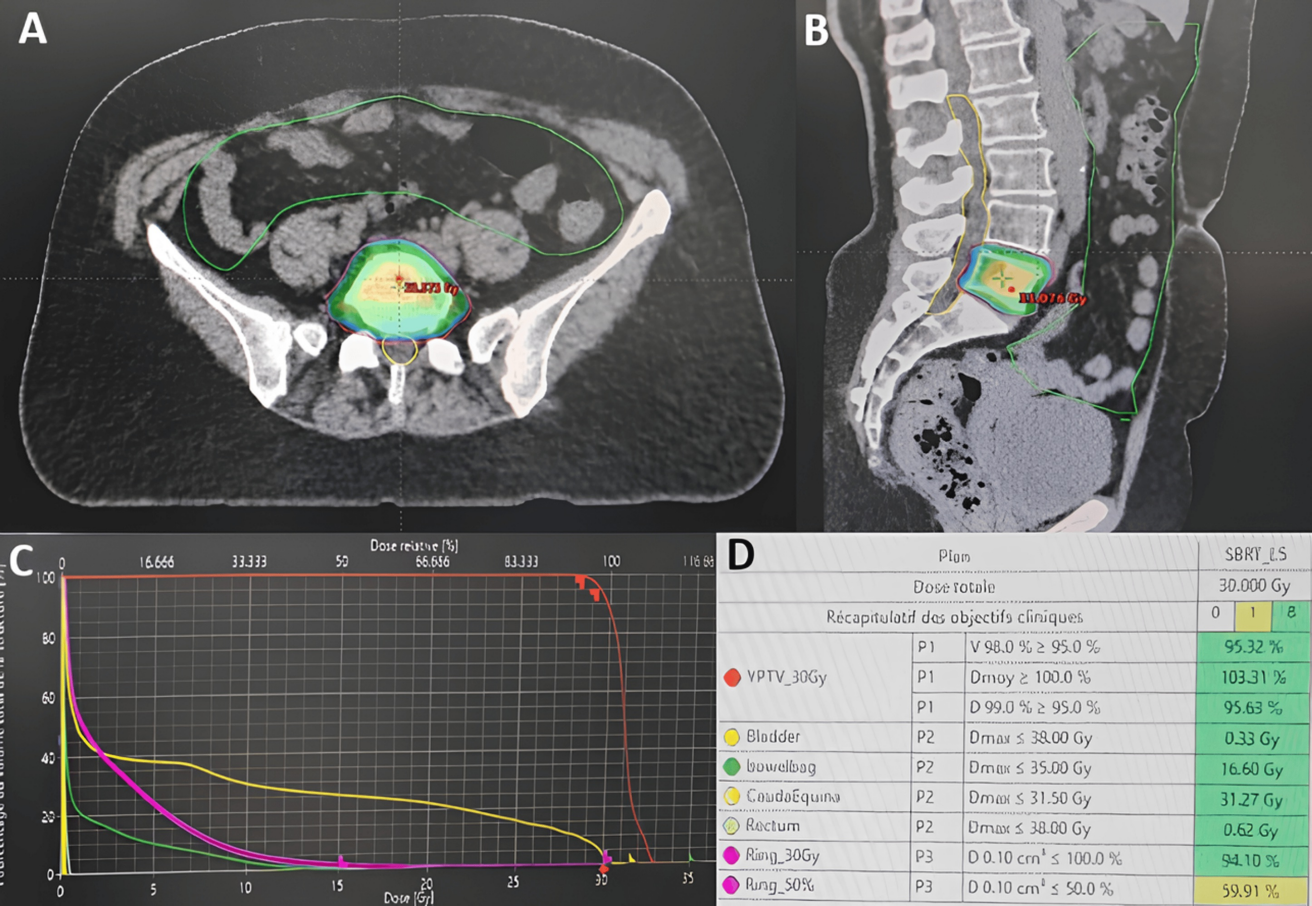
Stereotactic radiotherapy Axial (A) and sagittal (B) section of the dosimetric scan shows the distribution of isodoses at L5. The dose-volume histogram (C) illustrates the coverage of the planned target volume (PTV) and the dose received by organs at risk (bladder, rectum, cauda equina, bowel bag). The table (D) shows the dosimetric constraints with adequate coverage of the PTV.

After discussion in a multidisciplinary tumor board meeting, the patient underwent a right mastectomy with axillary lymph node dissection. Histopathological analysis revealed no residual invasive carcinoma (only benign fibrocystic changes), no Paget disease or in situ carcinoma, no lymphovascular or perineural invasion, clear surgical margins, and negative lymph nodes (0/4). The pathological response was classified as Chevallier grade 1 and Sataloff T-A/N-B. Adjuvant therapy included 3D conformal radiotherapy to the right chest wall (with bolus) and regional axillary and supraclavicular lymph nodes (total dose 50 Gy in 25 fractions over five weeks) (Figure 6), as well as stereotactic body radiotherapy to the L5 lesion (30 Gy in 5 fractions) ​​​​​​(Figure 7).

The patient received maintenance therapy with trastuzumab and pertuzumab for a total duration of two years. At the conclusion of this period, the patient is in complete remission, with no clinical or radiological evidence of disease recurrence.

## Discussion

Apocrine transformation of the mammary epithelium is a common phenomenon that can give rise to a spectrum of lesions, from benign changes to malignant neoplasms. Malignant lesions with this differentiation are primarily classified as apocrine ductal carcinoma in situ (ADCIS) or IAC [6]. The term 'apocrine carcinoma' was first introduced by Krompecher in 1916 and later popularized by Frable and Kay in 1968 [2]. However, specific histological criteria for its diagnosis were not defined until 2005, when Japaze et al. proposed that apocrine differentiation be diagnosed only if characteristic cells comprise at least 75% of the tumor [9,10]. These cells are large and well-defined, with abundant granular eosinophilic cytoplasm, large vesicular nuclei (nucleocytoplasmic ratio ≥ 1:2), and prominent nucleoli. In 2010, Vranic et al. refined these criteria by requiring that over 90% of the tumor cells display these apocrine features, accompanied by a distinct immunohistochemical profile, i.e., the absence of ER/PR and positive androgen receptor (AR) expression [11]. This strict definition helps distinguish 'pure' apocrine carcinoma from other breast cancers in which partial apocrine differentiation can be seen (approximately 30% of cases) [7,10].

Apocrine carcinoma of the breast is a sporadic and relatively uncommon disease, occurring predominantly in women between 50 and 79 years of age [12], as in our case. It has also been documented, albeit rarely, in male patients [13]. Apocrine carcinomas also exhibit varied imaging characteristics. On ultrasound, lesions may appear as hypoechoic cysts with papillary projections, as solid or complex masses, or as heterogeneous masses with irregular, ill-defined margins. On mammography, microcalcifications are frequently observed; however, some tumors present as oval nodules without calcifications [5].

The differential diagnosis primarily includes invasive ductal carcinoma (NST) and oncocytic (Hürthle cell) carcinoma. Invasive ductal carcinoma can occasionally exhibit a similar architectural appearance to apocrine carcinoma; however, cytological analysis allows differentiation. Apocrine cells are generally classified as either type A (characterized by abundant granular eosinophilic cytoplasm) or type B (foamy cytoplasm rich in lipid vacuoles). In our case, the majority of tumor cells displayed type A morphology. Distinguishing apocrine from oncocytic carcinoma relies on immunohistochemistry: apocrine carcinomas are typically ER/PR negative with positive AR and gross cystic disease fluid protein-15 (GCDFP-15) expression [7,10]. By definition, apocrine tumors express AR and GCDFP-15 while lacking ER, PR, BCL-2, and GATA3. Notably, GCDFP-15 positivity has been associated with a higher risk of local recurrence and distant metastasis [14]. Our case demonstrated the classic morphology and immunophenotype of a pure apocrine carcinoma. At the molecular level, apocrine carcinomas frequently harbor mutations in PIK3CA, PTEN, AKT, or TP53, whereas alterations in the MAPK pathway genes (KRAS, NRAS, BRAF) are less common. The HER2 gene amplification and protein overexpression are documented in approximately 30% of cases. By contrast, amplification of the EGFR gene is rare (~6%), despite frequent expression of the EGFR protein [4].

Because of the rarity of this tumor type, no standardized treatment protocol has been established. Treatment generally mirrors that of other breast carcinomas; surgery (either breast-conserving or mastectomy) with axillary lymph node dissection remains the cornerstone of therapy. The role of radiotherapy in apocrine carcinoma is not well defined, owing to the paucity of specific data. Endocrine therapy may be considered in 'non-pure' apocrine tumors that express hormone receptors (ER and/or PR) [1]. Overall, apocrine carcinoma has been reported to show limited responsiveness to conventional chemotherapy. However, in select cases, especially HER2-positive tumors, neoadjuvant chemotherapy combined with targeted anti-HER2 therapy has achieved complete pathological responses [3]. Our patient exemplifies this, having attained both radiological and pathological complete remission following neoadjuvant treatment. For patients with oligometastatic disease (defined as 1-5 metastatic lesions), stereotactic ablative radiotherapy (SABR) has emerged as a promising modality. In the Stereotactic Ablative Radiotherapy for the Comprehensive Treatment of Oligometastases (SABR-COMET) phase II trial, the addition of SABR to standard care conferred a median overall survival benefit of 22 months in patients with controlled primary tumors, compared to standard therapy alone [15].

The prognosis of apocrine breast carcinoma remains a subject of debate, partly due to the lack of uniform criteria in defining this rare entity [3]. Nonetheless, available data suggest that pure IAC may represent a distinct clinicopathological subtype with less aggressive behavior than high-grade NST breast carcinoma [16]. For example, Wu et al. compared outcomes of 366 patients with triple-negative apocrine carcinoma to those of 30,996 patients with triple-negative NST carcinoma and found that the apocrine group had a significantly more favorable prognosis, with a notable survival benefit from chemotherapy in that group [17]. Moreover, HER2-positive apocrine carcinomas have been reported to exhibit a less aggressive biological phenotype than their NST counterparts, resulting in better breast cancer-specific survival despite frequently low or absent ER/PR expression [18]. Several prognostic factors have been identified in the literature: high histologic grade (II-III), tumor size > 2 cm, and lymph node metastases are associated with worse outcomes, whereas breast-conserving surgery, hormone receptor positivity, and use of radiotherapy correlate with improved disease-specific survival [3].

## Conclusions

Apocrine breast carcinoma is a rare, distinct entity with unique morphological, immunohistochemical, and molecular characteristics, yet it remains poorly defined in current nosologies. This case illustrates the favorable outcome of a patient with HER2-positive apocrine breast carcinoma with oligometastatic disease, achieved through a personalized multimodal strategy combining chemotherapy, dual anti-HER2 therapy, surgery, conformal radiotherapy, and SABR. This case underscores the importance of individualized patient care guided by a multidisciplinary approach and the need to tailor treatment to the tumor’s biological characteristics and clinical course.
